# Supplementary material for: Stacking Model‐Based Classifiers for Dealing With Multiple Sets of Noisy Labels
Source: Biom J. 2025 Mar 12;67(2):e70042. doi: 10.1002/bimj.70042 (PMC11898607; doi:10.1002/bimj.70042)
Supplement: Supplementary file 1 — Supporting Information [file BIMJ-67-e70042-s001.zip › density-based-ensemble-model-main/competitors_real_data/netlab-master/netlab-master/help/mlphdotv.htm]

Netlab Reference Manual mlphdotv


# mlphdotv

## Purpose

Evaluate the product of the data Hessian with a vector.

## Synopsis

```
hdv = mlphdotv(net, x, t, v)
```

## Description

`hdv = mlphdotv(net, x, t, v)` takes an MLP network data structure
`net`, together with the matrix `x` of input vectors, the
matrix `t` of target vectors and an arbitrary row vector `v`
whose length equals the number of parameters in the network, and
returns the product of the data-dependent contribution to the Hessian
matrix with `v`. The implementation is based on the R-propagation
algorithm of Pearlmutter.

## See Also

`mlp`, `mlphess`, `hesschek`

---

**Pages:**
Index


---

Copyright (c) Ian T Nabney (1996-9)
